# Time‐Tailoring van der Waals Heterostructures for Human Memory System Programming

**DOI:** 10.1002/advs.201901072

**Published:** 2019-08-26

**Authors:** Huawei Chen, Chunsen Liu, Zuheng Wu, Yongli He, Zhen Wang, Heng Zhang, Qing Wan, Weida Hu, David Wei Zhang, Ming Liu, Qi Liu, Peng Zhou

**Affiliations:** ^1^ State Key Laboratory of ASIC and System School of Microelectronics Fudan University Shanghai 200433 China; ^2^ Institute of Microelectronics Chinese Academy of Sciences Beijing 100083 China; ^3^ School of Electronic Science & Engineering Nanjing University Nanjing 210093 China; ^4^ Shanghai Institute of Technical Physics Chinese Academy of Sciences Shanghai 20083 China

**Keywords:** decision‐making ability, human memory, time‐tailoring ability, van der Waals heterostructures

## Abstract

The human memory system plays an indispensable role in oblivion, learning, and memorization. Implementing a memory system within electronic devices contributes an important step toward constructing a neuromorphic system that emulates advanced mental functions of the human brain. Given the complex time‐tailoring requirement, integrating a human memory system into one system is a great challenge. Here, one van der Waals heterostructure with flexible time‐tailoring ability is demonstrated, which can meet the high requirement of human memory system programming. By stacking volatile and nonvolatile function layers, all basic memory types, including sensory memory, short‐term and long‐term memory are integrated into the device and the transition between these memory types are flexible. Moreover, decision‐making action and in situ result storage are also demonstrated. It is anticipated that the demonstrated time‐tailoring system will support the model of a human memory system.

## Graphical abstract

**van der Waals heterostructures with time‐tailoring ability** are developed for human memory system programming. Retention time can be tailored from milliseconds to kiloseconds. Thus, a human memory system with rich functionalities is implemented in this cell and the system also has a capability of decision‐making and in situ data storage.

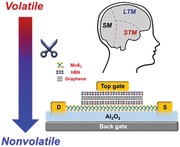

Human brain performs high efficiency task and massive data parallelism, where human memory is the advanced faculty by which information is encoded, stored, and retrieved. Information dissemination in human memory undergoes three typical memory types, including sensory memory, short‐term and long‐term memory.^1^ Specially speaking, short‐term plasticity (STP) performs a series of computations with a temporal strengthening of synaptic connections and dominates short‐term memory; Long‐term plasticity (LTP) provides a persistent modification of synaptic weight, thus underpinning long‐term memory;^2^ While sensory memory has been ignored or confused with short‐term memory,^3^, ^4^ which also plays an important role in instantaneous reaction. Besides, flexible transition among different memory types represents the information dissemination in a reasonable processing and it indicates a dynamic progress of received information involving forgetting, learning, and remembering.

To date, massive efforts have been devoted to exploring the realization of only STP or LTP, such as electric‐double‐layer transistors,^5^, ^6^, ^7^ resistive random access memory (RRAM),^3^, ^8^, ^9^ and phase change memory (PCM).^10^ Diffusive dynamics observed in memristors applies to STP,^11^, ^12^ such as paired‐pulse facilitation and paired‐pulse depression. Memristors with nonvolatile performance are exploited for the emulation of LTP,^13^, ^14^, ^15^ including long‐term potentiation, long‐term depression, and spike‐timing‐dependent plasticity. Dynamic features are very useful in describing short‐term memory and nonvolatile characteristics are powerful in demonstrating long‐term memory behavior. Although some works^3^, ^11^, ^16^, ^17^, ^18^ have been proposed to demonstrate concomitant STP and LTP, they still cannot integrate all memory types (especially sensory memory) with corresponding function in one system because of a lack of flexible time‐tailoring ability. Therefore, integrating different retention time and modulating the temporal to persistent memory transition are the important keys for human memory programming. Works about the integration of whole memory system with rich functionalities are still blank.

In consideration of the demand for complex time‐tailoring ability, 2D materials could offer one suitable solution.^19^, ^20^, ^21^, ^22^, ^23^ In fact, the unique structures of 2D materials with a dangling‐bond‐free lattice characterized by van der Waals forces between neighboring layers promise a combination of 2D materials in a designed sequence without any lattice marching constraints.^24^ The abundant characteristic of 2D materials, from insulators, semiconductors to conductors, also opens up the possibility of heterogeneous integration.^25^ Therefore, the stackable property and abundant characteristics can provide easier path to integrate layers with different retention time,^26^ and thus the complex time‐tailoring requirement of human memory system can be meet.

Here, we demonstrate single van der Waals heterostructure for complex time‐tailoring requirements of human memory system. By fully exploiting the unique property of 2D materials, we have designed two charge storage layers for an integration of volatile and nonvolatile performance. Therefore, the heterostructure successfully implements a human memory system, including sensory memory, short‐term and long‐term memory with the time scale ranging from milliseconds to kiloseconds. Rich functionalities, such as oblivion, memory transition, memorization‐level potentiation and depression, have been demonstrated. Furthermore, our heterostucture also has a capability of decision‐making with logic operation and stores the result in situ.

The external stimuluses are registered in sensory memory as instantaneous reaction to the world, as shown in **Figure** **1**a. The selected information transmits to short‐term memory and is supposed to decay and disappear completely. A big difference is that retention time in sensory memory is typically in the region of 200 to 500 ms while that in short‐term memory is usually less than 1 min. The information in short‐term memory after rehearsal converts to long‐term memory, and then the information could maintain for a long time. The received information fading away with time is defined with a process of “forgetting” and we can describe the process with “remembering” when the information is finally stored. In neuroscience, the synapse is a functional junction between two neurons and transmits signals through a tuning of synaptic weight. The synaptic plasticity can be mainly divided into STP and LTP. Therefore, different states of synaptic plasticity are analogous to different memory types in human memory system, in which short‐STP (S‐STP), long‐STP (L‐STP), L‐STP to LTP and LTP represents sensory memory, short‐term memory, short‐term memory to long‐term memory and long‐term memory, respectively.

**Figure 1:**
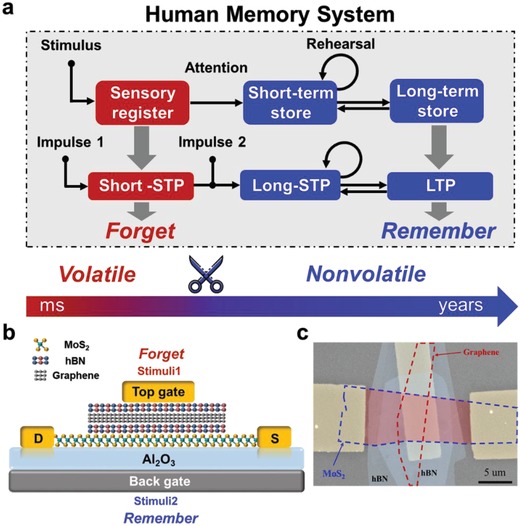
Human memory system and device characterization. a) Proposed human memory system with three memory types, including sensory memory, short‐term and long‐term memory. The retention time in human memory system ranges from millisecond to years, indicating an integration of volatile and nonvolatile characteristic in human brain. Three memory types show similarity in synapse plasticity with different retention time. b) Schematic structure of the device. The van der Waals heterostructure composed of MoS_2_/hBN/graphene/hBN layers. Top gate operation dominates the volatile characteristic and back gate operation dominates the nonvolatile characteristic. c) A false‐color SEM image of the device. The scale bar is 5 µm.

Schematic structure of the device is shown in Figure 1b. MoS_2_ flakes were used as the channel material. The Al_2_O_3_ layer and the hBN/graphene/hBN heterojunction were both employed as the charge storage stacks. The silicon substrate served as an extended back gate, defined as *V*_bg_, and the electrode on the hBN flakes was defined as *V*_tg_, which provides another electrostatic control over the device. Figure 1c presents a false‐color scanning electron microscope (SEM) image of the van der Waals heterostructure. The thicknesses and components have been further confirmed by atomic force microscopy (AFM) and Raman mapping (Figures S1 and S2, Supporting Information), respectively. The hBN/graphene/hBN stack was transferred onto MoS_2_ by a standard transfer method (Figure S3, Supporting Information).^27^

Time‐tailoring ability of the heterostructure depends on designed volatile and nonvolatile function layers with tailoring memory time. In fact, the thickness of tunneling layers and memory working modes are two key factors to memory characteristic:^28^, ^29^ The thickness depends on volatile or nonvolatile performance while memory working modes decide erasing/programming polarity. Toward memory working mode, channel injection mode and electrode injection mode are included. When the device is in channel injection mode,^28^ electrons from channel are trapped in trapping‐layer under positive voltage, which would lead to clockwise memory window; In electrode injection mode, electrons from electrode are trapped in trapping‐layer under negative voltage and cause anticlockwise memory window. The device exhibits volatile performance when operated with top gate. The transfer characteristics are studied when *V*_tg_ sweeps from −6 to 6 V and returns to −6 V (**Figure** **2**a). The on/off ratio is estimated to be 10^6^, and a clear anticlockwise hysteresis window of 2 V is observed. The transfer curve also shows high repeatability after 100 *I*–*V* dual sweeps. As shown in Figure 2c, the programmed/erased states return to its initial state in 10 s after applying *V*_tg_ = ±6 V with a pulse duration of 2 s, illustrating typical volatile characteristic. We assume that volatile performance contribute to van der Waals heterostructure of hBN/graphene/hBN. We illustrate this phenomenon by energy band diagrams in detail (Figure 2e). When the device is in top gate operation, tunneling layer (7 nm) is close to top electrode and it works in electrode injection mode. Electrons from top electrodes are injected into graphene layer through hBN under a negative *V*_tg_. Negative (positive) *V*_tg_ that causes a positive (negative) shift of threshold voltage leads to the anticlockwise hysteresis. However, tunneling electrons (holes) cannot be stored in graphene for a long period due to adopted thin tunneling layer. Control experiments have been conducted to verify that hBN/graphene/hBN should be responsible for volatile performance, considering that the hysteresis loop is a normal phenomenon in 2D field effect transistors (FETs),^30^, ^31^, ^32^ which involves several possible explanations (Figure S4, Supporting Information). The device composed of MoS_2_/hBN showed almost no hysteresis loop with top gate operation (Figure S5, Supporting Information). Gate voltage showed only electrostatic control over MoS_2_ flake without any relaxation process, which addressed the necessity of a graphene layer and an hBN layer.

**Figure 2:**
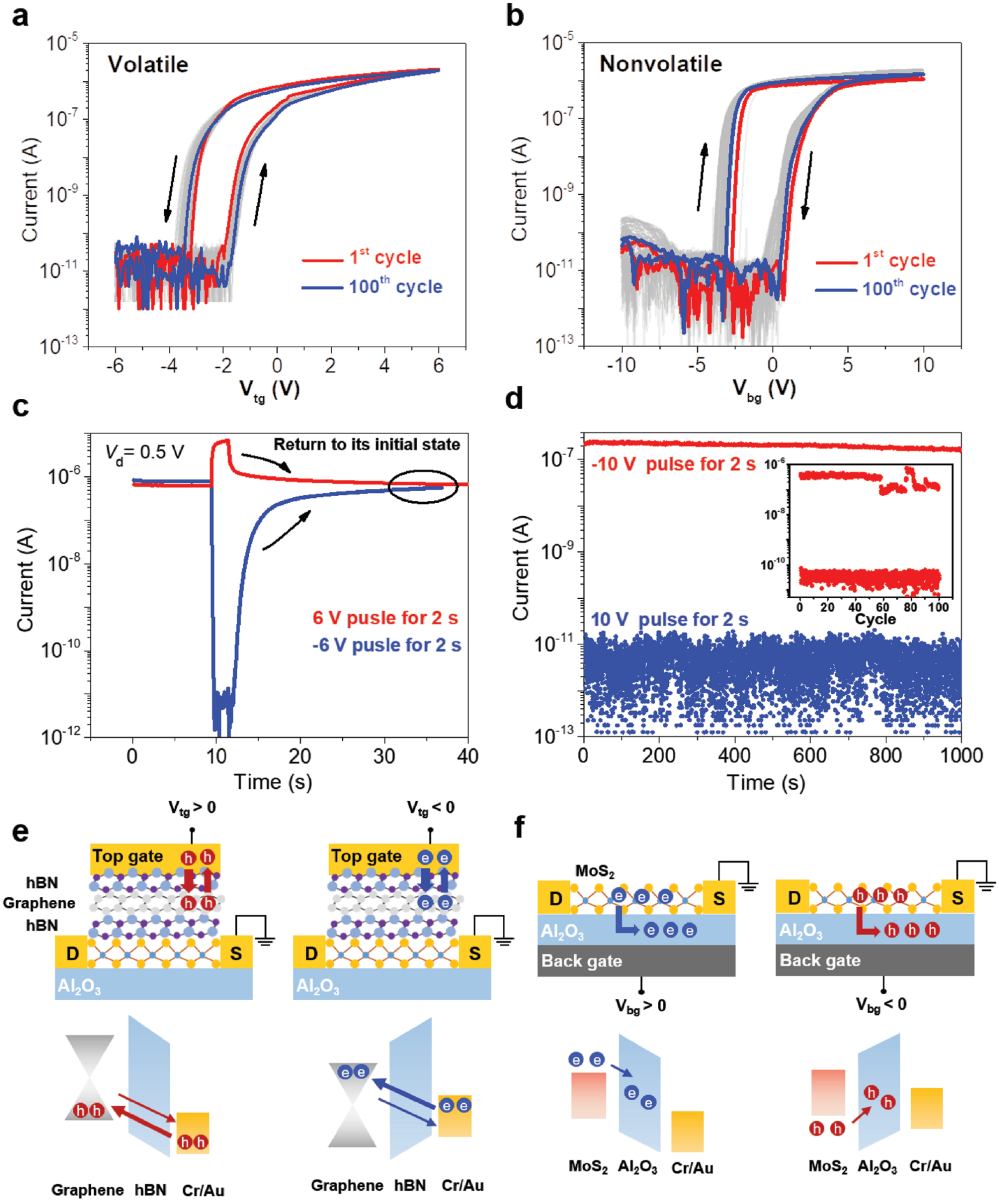
Time‐tailoring ability of the van der Waals heterostructure. a) Typical *I*_ds_–*V*_tg_ characteristics at the 1st (red) and 100th (blue) cycle when *V*_ds_ = 0.5 V. It exhibits an anticlockwise loop of 2 V and volatile characteristic. b) Typical *I*_ds_–*V*_bg_ characteristics at the 1st (red) and 100th (blue) cycle when *V*_ds_ = 0.5 V. It exhibits a large memory window of 4 V and nonvolatile characteristic. c) Volatile characteristic when operating with top gate and different states return to its initial states in 10 s. d) Nonvolatile characteristic when operating with back gate and different states remain stable in 1000 s. The endurance property over 100 cycles is shown as an inset. e) Energy band diagram of volatile characteristic illustration. f) Energy band diagram of nonvolatile characteristic illustration.

The heterostructure shows nonvolatile performance with back gate operation, by utilizing the capability of ALD‐grown high‐κ dielectric. To explore the data storage capability of our device, the transfer curve is first investigated (Figure 2b). *V*_bg_ starts with a forward sweep followed by a backward sweep under a *V*_ds_ bias of 0.5 V when *V*_tg_ is grounded. A memory window of 4 V can be observed when the maximum *V*_bg_ is 10 V. To assess the memory retention ability, we apply *V*_bg_ = ±10 V with a pulse duration of 2 s. It shows a programmed/erased state ratio over 10^4^ under a *V*_ds_ of 0.5 V and two states maintain stable over 1000 s (Figure 2d). The endurance over 100 cycles is also shown as an inset. Compared with other 2D memory devices,^33^, ^34^, ^35^ the device shows a rather low operation voltage of 10 V which is due to the 23 nm thick Al_2_O_3_ layer. Here, the high‐κ layer functions as both a charge‐trapping layer and a charge‐blocking layer.^36^ The device with back gate operation works in channel injection mode and the mechanism is illustrated via the energy diagram (Figure 2f). Under a high positive *V*_bg_, electrons in MoS_2_ flakes tunnel into Al_2_O_3_ layer, and the accumulation of electrons in the Al_2_O_3_ layer could partially screen the electric field from the back gate, causing a positive shift in the threshold voltage. The positive shift in the threshold voltage correspondingly results in a lower current level, which is defined as programmed state. In erase operation, a negative *V*_bg_ is required. The trapped electrons tunnel back to the MoS_2_ channel from the Al_2_O_3_ layer, causing the threshold voltage to move along the negative direction. The current level becomes higher than that in the initial state, and this condition is defined as the erased state.

In fact, the integrating of volatile and nonvolatile performance in the same cell has been a great challenge. Time‐tailoring ability has been built into our heterostructure via designing different charge storage layers by exploiting unique property of 2D materials. Furthermore, memory retention time can be dynamically modulated by each gate terminal.^37^ If channel materials are replaced with ambipolar 2D flakes, such as black phosphorus or WSe_2_, then the channel polarity can be switched reversibly between holes and electrons.^36^ The reproducibility of van der Waals heterostructures with time‐tailoring ability is also demonstrated (Figure S6, Supporting Information).

As discussed earlier, complex time‐tailoring requirement and memory type transition are the important keys in human memory system programming. Complex time‐tailoring requirement determines high biological similarity of human memory system programming in retention time, as shown in **Figure** **3**a. Memory type transition represents the information dissemination in a reasonable processing and can be listed as follow: a) the information in sensory memory converts to short‐term memory by “attention.” b) The information can be transferred between short‐term and long‐term memory by “rehearsal” or “retrieval.” c) The information in sensory memory cannot convert to long‐term memory directly.

**Figure 3:**
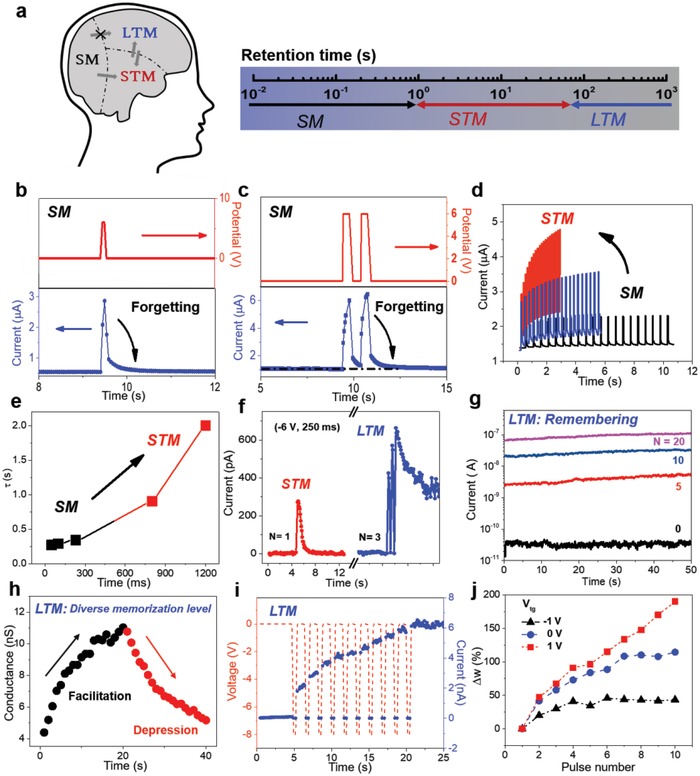
Human memory system programming. a) Two key points when executing human memory system programming: complex time‐tailoring requirement and flexible transition among different memory types. b) Measured current triggered by the stimulus (top gate, 6 V, 100 ms) decays to its initial state in 200 ms, indicating the forgetting process in sensory memory (SM). c) Measured current triggered by two successive stimulus (top gate, 6 V, 300 ms) with a time interval of 500 ms. d) Flexible transition from SM to short‐term memory (STM) when increasing the stimulus frequency. However, SM cannot convert to long‐term memory (LTM). e) The plot of retention time and spike width and a flexible transition from SM to STM could be observed. f) Measured current triggered by the stimulus (back gate, −6 V, 250 ms) decays to its initial state in 1 s, indicating the forgetting process in STM. The transition from STM to LTM is demonstrated when input numbers increase from one to three. g) Retention time of different memorization states when applying different number (*N* = 0, 5, 10, and 20) of spikes (back gate, −6 V, 500 ms). h) The facilitation and depression effect on memorization level from electrical stimulus with different spike (back gate, −6 V, 400 ms, 3 V, 300 ms). i) The dynamic response of facilitation effect on LTM with ten successive spikes (−8 V, 300 ms) and top gate is biased at 1 V. j) The modulation effect of top gate on LTM programming. Positive top gate exhibits an obvious facilitation on long‐term change while negative bias suppresses this change.

Human memory system programming is successfully demonstrated here by our approach. For our structure, back gate and top gate terminals are utilized as inputs for programming different memory types. We employ MoS_2_ channel with source/drain electrodes as current‐monitoring output. The measured current value and dynamic response represent the human memory programming condition. Figure 3b displays a typical behavior in sensory memory. The current characteristic was triggered by a spike (6 V, 100 ms) from top gate with a *V*_ds_ bias of 0.5 V. Measured current rapidly reaches a peak value of ≈2.86 µA and gradually returns to its initial state of ≈0.56 µA in 500 ms. The current response reflects a temporal response toward the external stimulus and decays to its initial state in 200 ms, indicating a similarity to the process of “forgetting” in sensory memory. The current value can be enhanced by following a second spike in short time. Figure 3c shows the response when evoked by two successive spikes (6 V, 300 ms) with a time interval of 500 ms and the current decays to initial state in 500 ms. Two different current peaks (A1 and A2) are observed in this condition where A2 is larger than A1 and current index (A2/A1) as a function of the interval time (Δ*t*) is shown in Figure S7 (Supporting Information). Perception intensity in sensory memory can be enhanced by increasing spike number and sensory memory converts to short‐term memory. Shown in Figure 3d, sensory memory turns into short‐term memory with stronger intensity and longer memory time when the stimulus frequency increase from 2 to 10 Hz. It is worth noting that sensory memory cannot convert to long‐term memory, which is desirable in human memory system because of the volatile characteristic when employing top gate operation. The relaxation process was fitted by an exponential function, which was used to describe the forgetting function in psychology^38^

(1) $$I_{t}=I_{0}\;+A\exp\left(-t/\tau \right)$$

where *I*_0_ represents the stable current, *A* is a prefactor, and τ is a relaxation time constant. Figure 3e shows a plot of spike duration‐dependent relaxation time. Constant τ shows positive correlation with spike duration, which also indicates a transition from sensory memory to short‐term memory. As stated earlier, the device characteristic can be modulated via top gate and back gate terminals respectively. The energy dissipation for the device in volatile regime is estimated to be 40 nJ per spike when back gate bias is zero. A low energy consumption of ≈64 pJ per spike is obtained when utilizing negative back gate bias and decreasing *V*_ds_. The relationship between power consumption and back gate voltage has been studied in Figure S8, Supporting Information.

The van der Waals heterostructure with back gate operation executes programming on other memory types. It is all known that learning can be described as the ability to acquire new information where short‐term memory converts to long‐term memory, while remembering presents the mechanism that the knowledge is stored for a long time in long‐term memory. Shown in Figure 3f, triggered current (270 pA) decays to the initial state (1 pA) in 2 s after applying a spike (−6 V, 250 ms), illustrating forgetting behavior in short‐term memory. The retention time in short‐term memory is longer than that in sensory memory, due to the time‐tailoring ability of our heterostructure, and this ability satisfies the high demand of human memory programming for different memory time. Short‐term memory at this stage has the possibility to convert to long‐term memory and this transition illustrates the process of “learning” via rehearsal. Stimulus with different strengths (number, amplitude, and duration) are applied to explore the transition. As seen in Figure 3f, the rest current increases from 1 to 300 pA after applying three successive pulses (−6 V, 250 ms) and retention time has been extended. The pulse amplitude and duration also facilitate this process (Figure S9, Supporting Information). Such transition can be explained by the charge‐trapping effect.^39^ When a small bias applies at back gate, electrons are trapped by shallow traps in Al_2_O_3_ near the interfacial for a short time. When a higher bias is utilized, electrons would tunnel into deep traps and be stored for a longer time. Meanwhile, MoS_2_ is highly sensitive to charged states in Al_2_O_3_ layer due to its ultrathin body and thus electrical properties is easily affected.

The encoded information finally enters into long‐term memory and it involves a series of activities about long‐term changes. The retention time of different memorization states has been investigated. Diverse numbers (*N* = 0, 5, 10, and 20) of spikes (−6 V, 500 ms) are applied to the heterostructure, starting from the same initial state. The current is measured under a *V*_ds_ of 0.5 V after 5 s. Various states show a clear difference after 50 s, indicating the nonvolatile characteristic with back gate operation (Figure 3g). The modulation of spike numbers on long‐term change has also been demonstrated. 10 successive pulses (−6 V, 400 ms) are applied and followed by another ten successive pulses (3 V, 300 ms) with an interval of 500 ms, while the conductance of our device increases from 4.3 to 11.02 nS and decreases to 5.16 nS correspondingly (Figure 3h). The change of the conductance reflects strong facilitation and depression effect on memorization level from electrical stimulus with different spike numbers and polarity. The facilitation effect can be further modulated by applying a top gate bias. Figure 3i shows the dynamic response when the heterostructure is simulated by 10 successive pulses (−8 V, 300 ms) and top gate voltage is biased at 1 V. The facilitation effect has been extracted and plotted with pulse number and top gate voltage in Figure 3j. The positive top gate bias exhibits an obvious facilitation on long‐term change, while the negative bias suppresses the change. This emphasizes the tunability of our human memory model in long‐term memory programming.

Decision‐making ability consists of different logic operation, making up an important functionality in human brain.^40^ Toward all sorts of stimulus from the world, neurons should make decisions to ensure that correct response is obtained. Human memory system programming studied before reveals the response toward stimulus in asynchronous states, while decision‐making action indicates a logical process stimulated by two different spikes in synchronous states and the correct response could be stored. The heterostructure can function as a logic gate, adding decision‐making ability in our human memory system. Primary “OR” logic function is demonstrated in **Figure** **4**a, by utilizing back and top gate terminal separately as input A and input B. Negative (positive) input of 2 V is defined as logical 0 (1) and output currents have two states, which are denoted as logical 0 (low‐level current) and logical 1 (high‐level current). Then output currents are monitored over time with a *V*_ds_ of 0.5 V when inputs A and B vary across all four possible logic combinations: (0,0), (0,1), (1,0), and (1,1). The OR gate here generates a high‐level output current if either input is in logical 1 state. The result shows a high ratio over 10^3^ in two different logical states, which is much higher than that in reported literatures.^5^, ^41^, ^42^ Endurance of logic operation over 100 cycles is shown in Figure 4b, indicating the stable working state of our device. Figure S10 (Supporting Information) shows logic operation in one period. It is worth noting that the logic working mechanism is different from that of traditional Si transistors.^43^ For a traditional silicon transistor, at least two transistors are needed to construct “OR” logic gate. By our approach, only one device is employed and its bottom surface and top surface are controlled by top gate and back gate, respectively. Only when both gate give a low input signal, the device would output low current and thus “OR” is obtained. The mechanism has been discussed in detail in Figure S11, Supporting Information.

**Figure 4:**
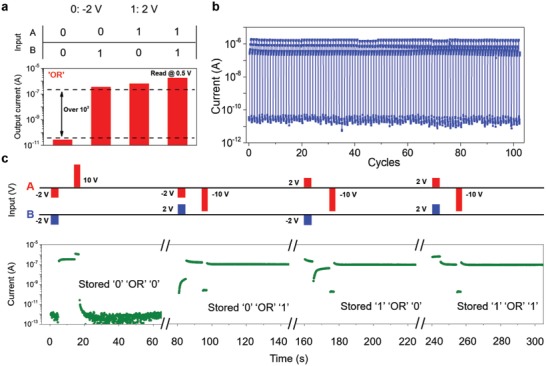
Decision‐making ability and in situ data storage. a) Demonstration of “OR” gate with a high ratio over 10^3^, indicating a simple decision‐making ability. b) Endurance property of “OR” operation over 100 cycles. c) Dynamic response of “OR” logic operation and stores the result in situ.

Furthermore, the result can be stored in situ, illustrating a process of “remembering” after decision‐making action in human memory system. A single operation can be divided into four steps: logic operation (signal amplitude, 2 V), waiting time, memory operation (signal amplitude, 10 V), and data storage monitoring (Figure S12, Supporting Information). The van der Waals heterostructure is initialized to state “1” at first. When (0,0) is input, OUT‐0 is achieved; Logic‐0 is stored in the same device by applying a memory voltage of 10 V from back gate. When other combinations including (0,1), (1,0), and (1,1), OUT‐1 is achieved and logic‐1 could be stored with a memory voltage (−10 V) from back gate (Figure 4c). Thus, a function of decision‐making and in situ data storage are demonstrated completely. A detailed comparison with other work is provided in Note S1 in the Supporting Information.

In this work, we demonstrate van der Waals heterostructures with time‐tailoring ability for human memory system programming. By exploiting unique characteristics of 2D materials, three memory types, including sensory memory (≈millisecond level), short‐term memory (≈second level) and long‐term memory (≈kilosecond level) have been integrated into our system. Rich functionalities based on the memory system, including oblivion, memory transition, memorization‐level potentiation, and depression are demonstrated. Furthermore, our human memory system also has a capability of decision‐making with logic operation and stores the result in situ. The demonstrated van der Waals heterostructure makes itself an appropriate candidate for human memory system programming.

## Experimental Section

*Device Fabrication*: The MoS_2_, hBN, and graphene flakes were obtained by mechanical exfoliation on a highly doped silicon substrate with a 23 nm Al_2_O_3_ high‐κ oxide layer. All 2D materials were bought from HQ‐graphene and a high‐κ oxide layer was deposited on the bare silicon wafer by ALD technology. The materials with proper thicknesses were identified under microscope and were stacked into the designed van der Waals structure (MoS_2_/hBN/graphene/hBN) by the polyvinyl alcohol (PVA) transfer method. The electrodes were defined by standard electron beam lithography, and Cr/Au metal films of ≈5 nm/30 nm in thickness were deposited using electron‐beam evaporation. The patterns on hBN and MoS_2_ were defined as the top and source/drain electrodes, respectively. The lowest silicon layer functioned as the bottom electrode.

*Structural Characterization*: The morphology of the exfoliated materials was identified by optical microscopy (9XB‐PC). The morphology of the device structure was confirmed by SEM (Hitachi SU1510). The thickness was determined by AFM (Bruker Veeco Multimode 8).

*Electrical Measurement*: The fabricated devices were measured using a probe station (Cascade1100) and a semiconductor device parameter analyzer (Keithley 4200SCAS). The DC signals and voltage pulses were both generated using a Keithley 4200 source/monitor unit (SMU) in 4200. All the measurements were tested at room temperature.

## Conflict of Interest

The authors declare no conflict of interest.

## Supporting information

Supplementary
