# Supplementary material for: Time‐Tailoring van der Waals Heterostructures for Human Memory System Programming
Source: Adv Sci (Weinh). 2019 Aug 26;6(20):1901072. doi: 10.1002/advs.201901072 (PMC6794622; doi:10.1002/advs.201901072)
Supplement: Supplementary file 1 — Supplementary [file ADVS-6-1901072-s001.pdf]

Copyright WILEY-VCH Verlag GmbH & Co. KGaA, 69469 Weinheim, Germany, 2019.

ADVANCED SCIENCE
Open Access

Supporting Information

for *Adv. Sci.*, DOI: 10.1002/advs.201901072

Time-Tailoring van der Waals Heterostructures for Human Memory System Programming

Huawei Chen, Chunsen Liu, Zuheng Wu, Yongli He, Zhen Wang, Heng Zhang, Qing Wan, Weida Hu, David Wei Zhang, Ming Liu, Qi Liu, and Peng Zhou**

Supporting Information

Time-tailoring van der Waals heterostructures for human memory system programming

Huawei Chen[1], Chunsen Liu[1], Zuheng Wu[2], Yongli He[3], Zhen Wang[4], Heng Zhang[1], Qing Wan[3], Weida Hu[4], David Wei Zhang[1], Ming Liu[2], Qi Liu[2*] and Peng Zhou[1*]

[1]State Key Laboratory of ASIC and System, School of Microelectronics, Fudan University, Shanghai 200433, China

[2]Institute of Microelectronics, Chinese Academy of Sciences, Beijing 100083, China

[3]School of Electronic Science & Engineering, Nanjing University, Nanjing 210093, China

[4]Shanghai Institute of Technical Physics, Chinese Academy of Sciences, Shanghai 20083, China

[*]Email: pengzhou@fudan.edu.cn

[*]Email: liuqi@ime.ac.cn

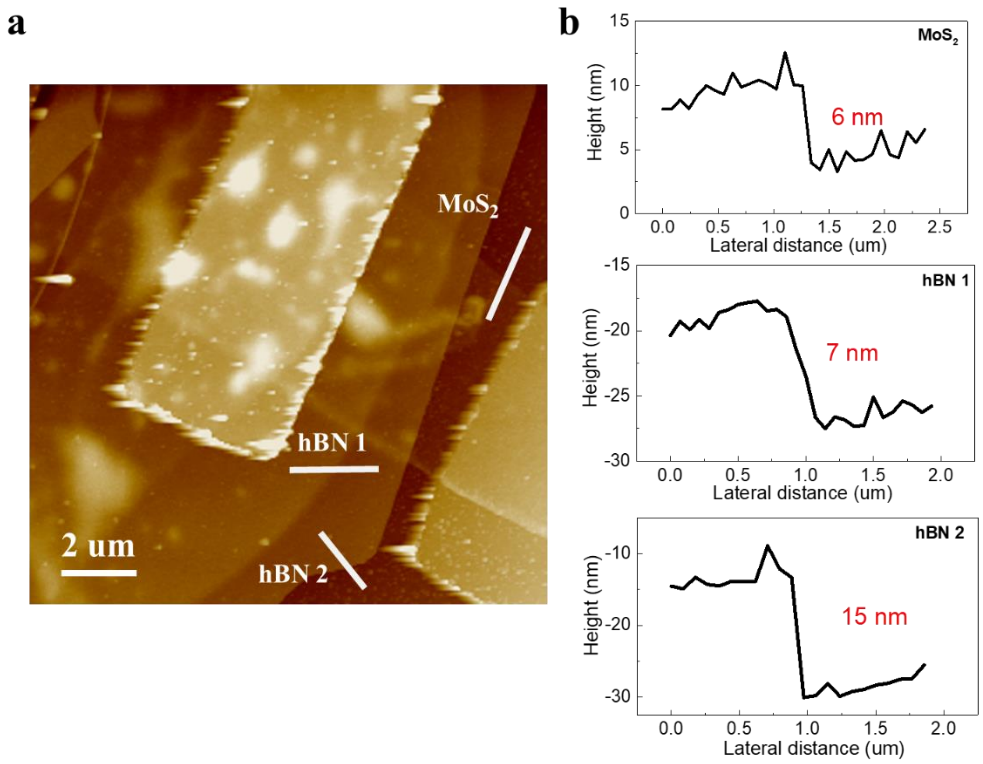

Figure S1. AFM images of the van der Waals heterostructures. a) AFM image of the device. White bubbles are the air gap, which are formed during the transfer method. The scale bar represents 2 μm. b) Height profiles of MoS_2, hBN1 and hBN2 flakes, are estimated to be 6, 7 and 15 nm.

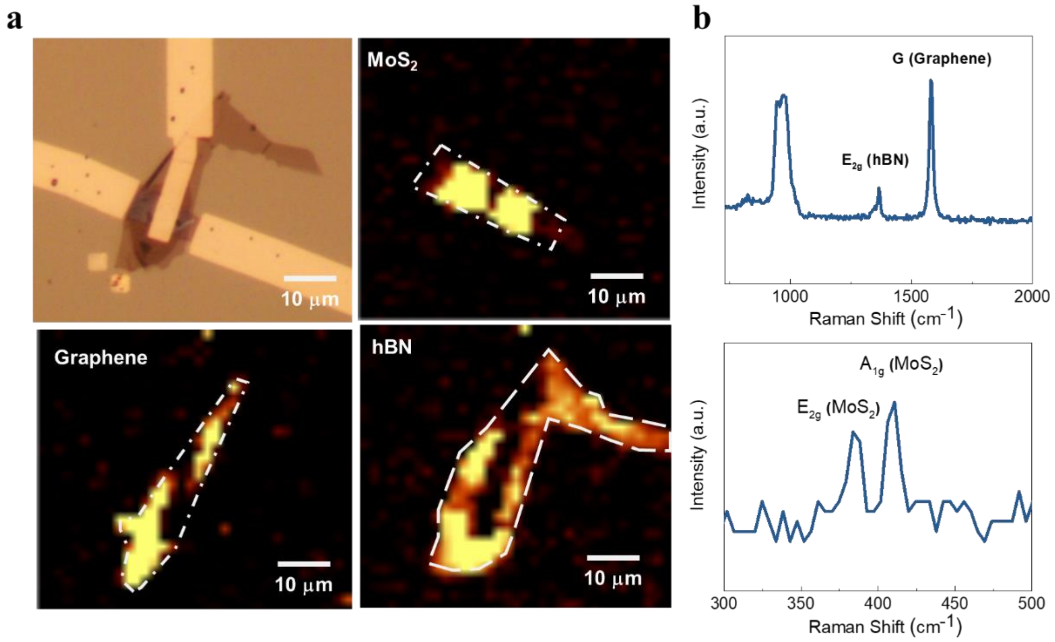

Figure S2. Raman mapping of the van der Waals heterostructures. a) The components of the devices are confirmed by Raman mapping. The scale bar represents 10 μm. b) Characteristic peaks of each materials for Raman mapping during the test.

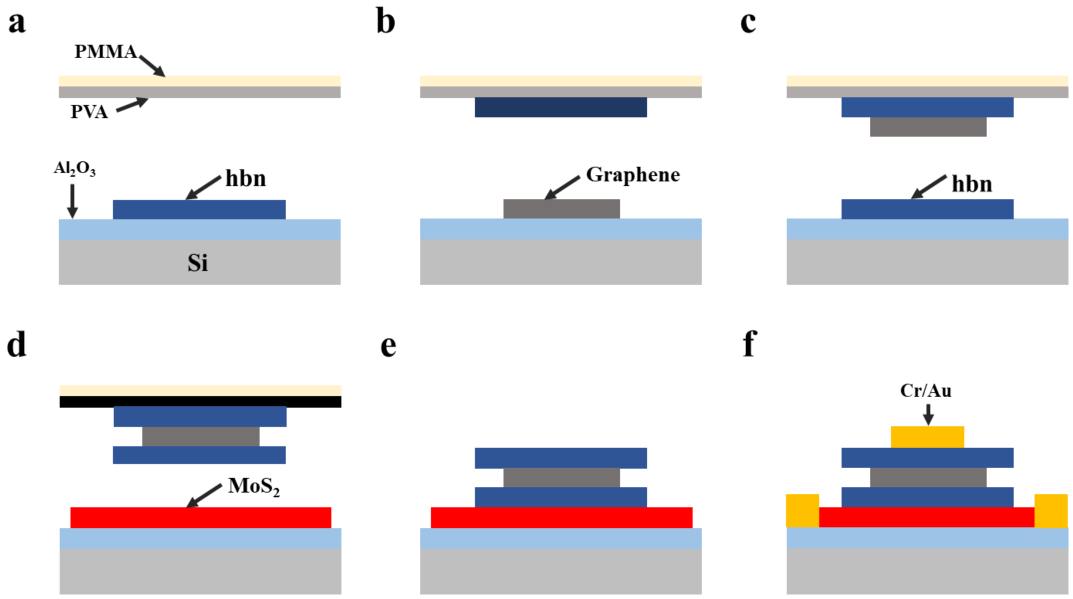

Figure S3. The fabrication process of the van der Waals heterostructures. a) hBN flakes were firstly transferred to PDMS/PVA films. b) Graphene flakes were then transferred under hBN flakes. c) Another hBN flakes were transferred under graphene flakes. d) PDMS/PVA with hBN/graphene/hBN was sticked on MoS_2 flakes and then PDMS was peeled off. e) The heterojunction was put in Deionized water and IPA successively for the dissolution of PVA. f) The electrodes were patterned in the corresponding area.

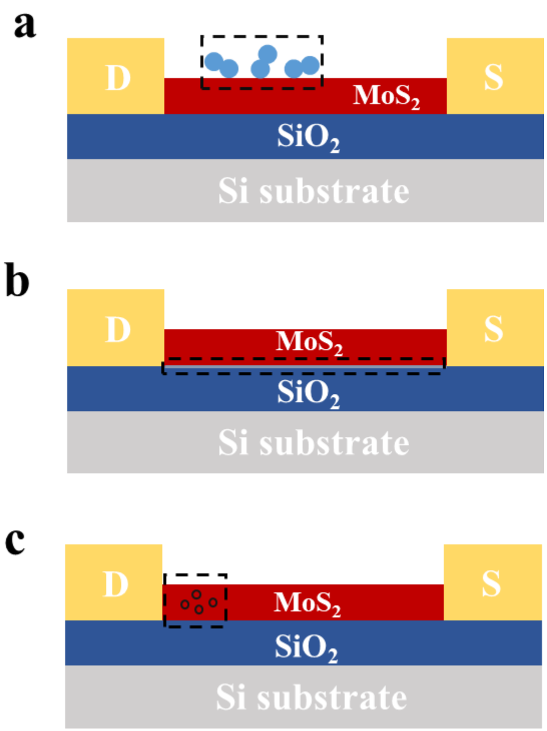

Figure S4. The proposed origin of hysteresis in MoS_2 FETs. a) Li et al [1,2] believed the adsorbed gas molecules such as H_2O and O_2 on the surface should be responsible for observed hysteresis. They thought the process of electron trapping and detrapping happened between the channel and the adsorbed gas molecules. b) Others [3,4] suggested the interface between gate oxide and channel materials was the main reason. There interface trap states are formed from the dangling bands. c) Shu at al [5]. thought the electron traps were attributed to the defects in channel materials.

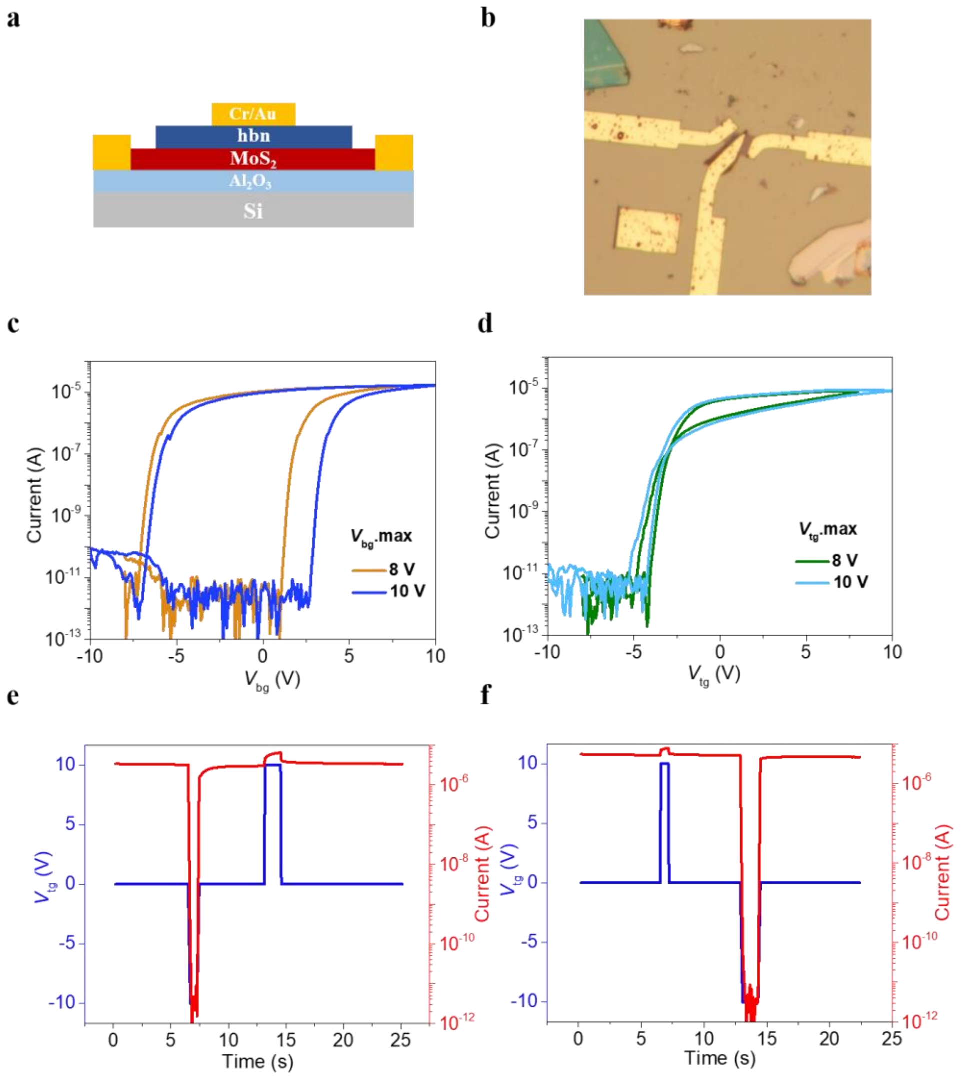

Figure S5. The control experiment and its corresponding electrical characteristics. a) The schematic structure of the device, which composed of MoS_2 and hBN without a graphene layer. b) The optical image of the device. c) The device was in back gate operation. The transfer curves of I_{ds}-V_{bg} exhibits a large memory window. d) The device was in top gate operation. The transfer curves of I_{ds}-V_{tg} exhibits almost no hysteresis loop. This indicates a clean interface between MoS_2 and hBN layers, which indirectly illustrates the importance of graphene layer for a memory window. e) and f) The retention behavior when applying positive and negative voltage successively. Top gate only provides electrostatic control over MoS_2

without any relaxation process.

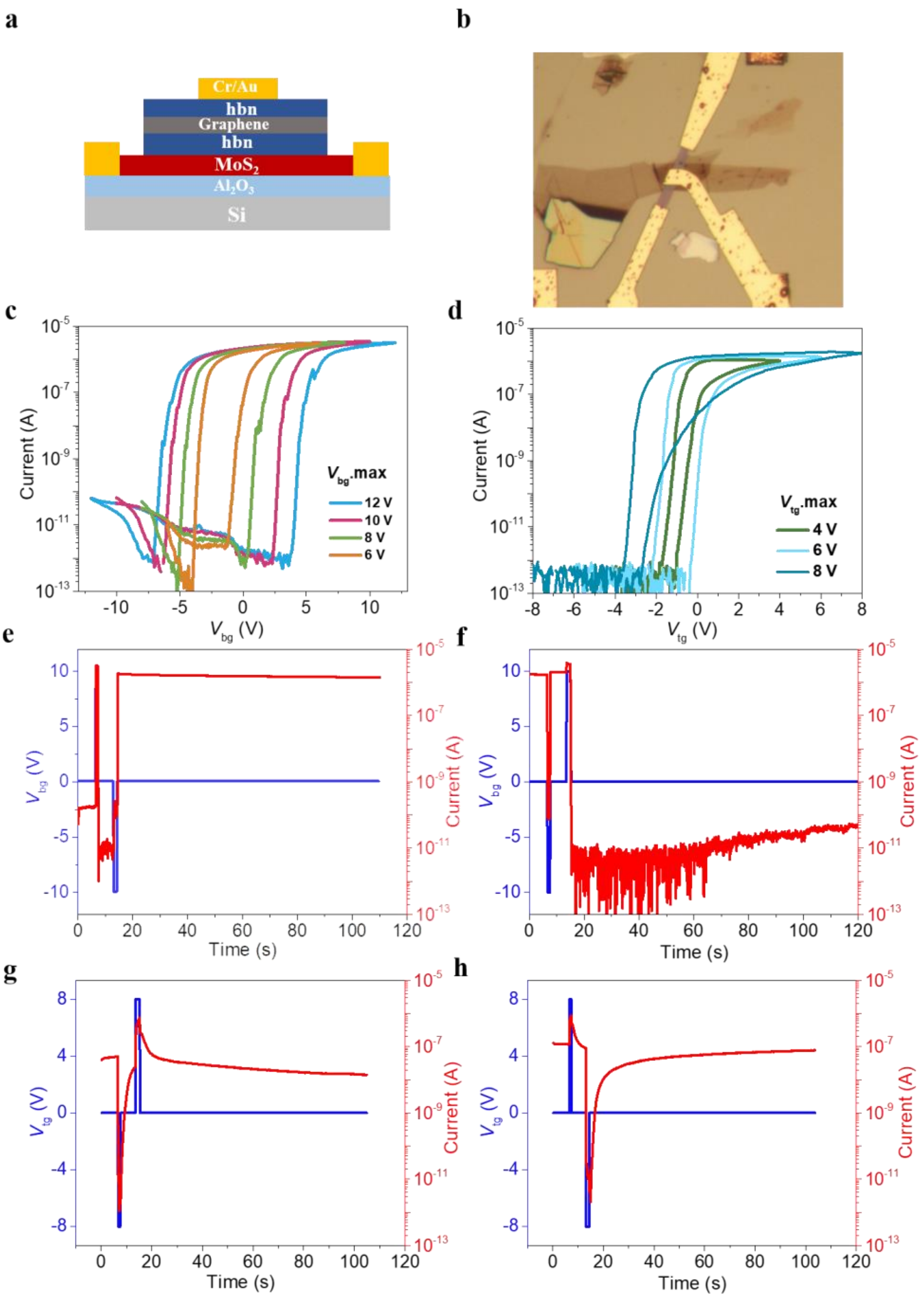

Figure S6. The reproducibility of the van der Waals heterostructures with time-tailoring ability. a) Schematic structure of another heterostructures. b) Optical image of the device. c) The device was in back gate operation. A large memory window can be observed. d) The device was in top gate operation. A large hysteresis loop can be observed. e) and f) Nonvolatile data retention performance when operated

with back gate. g) and h) Volatile data retention performance when operated with top gate.

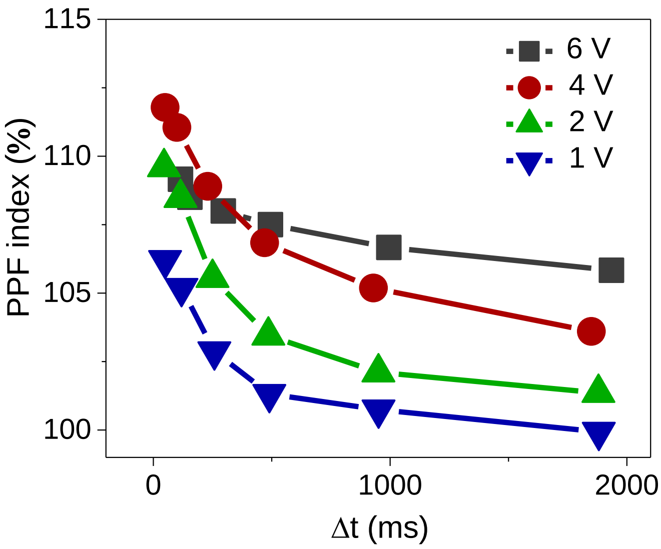

Figure S7. The index as a function of the interval time (Δt).

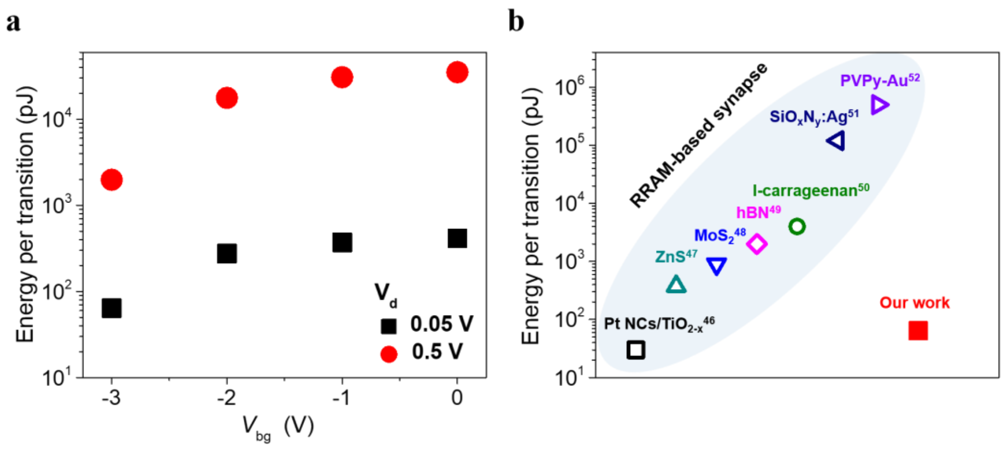

Figure S8. Energy consumption of the van der Waals heterostructures. a) Energy consumption modulated by back gate. The energy consumption per transition is estimated by the following equation:

$$W = V \int I_{peak}\, dt \qquad (1)$$

In the transistor-based synapses, V, I_{peak} and dt are source-drain voltage, pulse-triggered peak current and pulse duration time, respectively. The power consumption of our synaptic device is 40 nJ/spike when current value is read under no back gate voltage. One advantage of transistor-based synapses is the tunable current *via* gate voltage (tunable threshold voltage). When we decrease back gate voltage and source-drain voltage while keeping pulse amplitude at 1 V, a low energy consumption of ~64 pJ/spike is obtained. b) The comparison of energy consumption between our device and other RRAM-based devices. In RRAM-based devices, V, I_{peak} and dt are pulse voltage, pulse-triggered current and pulse width, respectively. Although switching speed of RRAM is on the ~ns level, applied pulse width when imitating synapse behavior usually is on the ~µs level. So, the energy consumption of our device is comparable to that of RRAM-based devices.

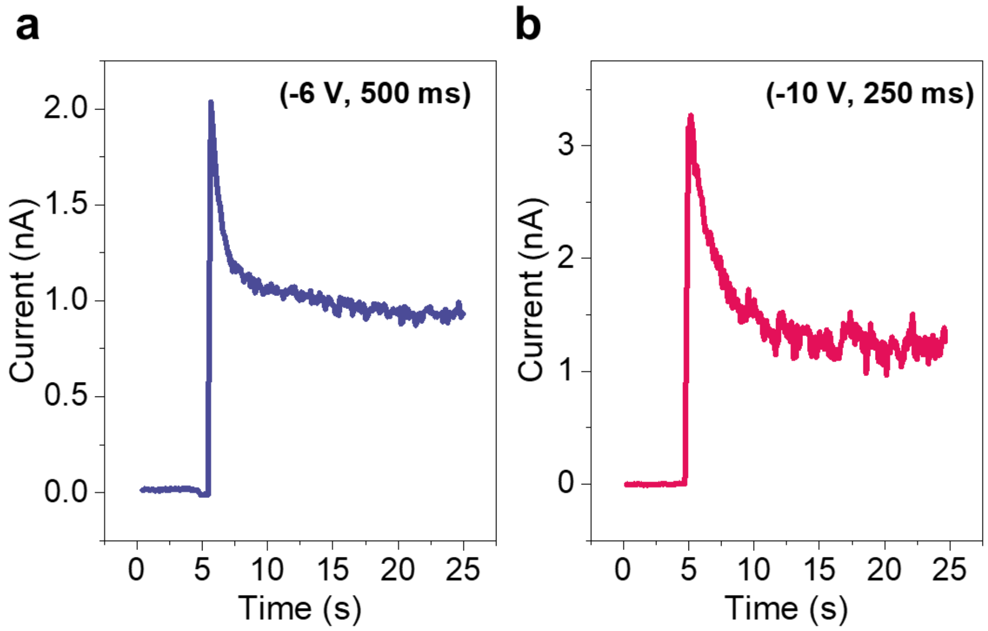

Figure S9. LTM emulation by changing duration and amplitude. a) When increasing the pulse duration from 250 ms to 500 ms and keeping the amplitude at 6 V, STM converts into LTM. b) When increasing the amplitude from 6 V to 10 V and keeping the duration at 250 ms, STM converts into LTM.

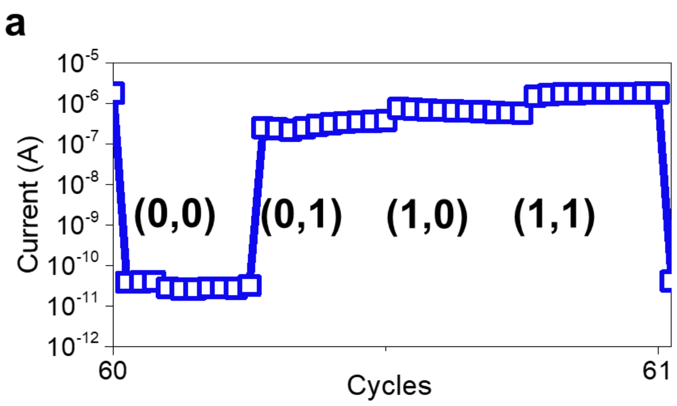

b

In A	In B	A OR B
0	0	0
0	1	1
1	0	1
1	1	1

Figure S10. Logic operation of the synaptic transistor. a) The dynamic process during one period. The result shows a high ratio over 10^3. b) The truth table of "OR" operation.

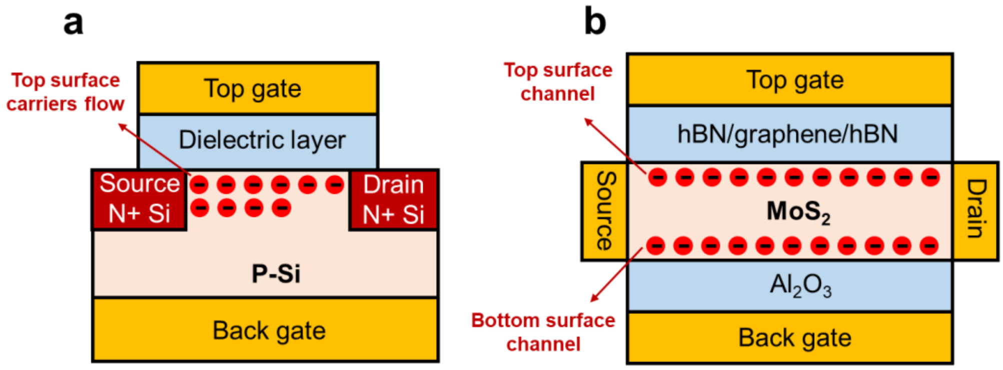

Figure S11. The working mechanism of silicon transistors and 2D transistors. For a traditional silicon transistor, at least two transistors are needed to construct ‘OR’ logic gate. By our approach, only one 2D transistor is employed. In traditional transistors, only top surface of channel has proper doping level to serve as carriers flow surface because of the intrinsic thickness of bulk silicon. It could be seen as one working channel. However, top surface and bottom surface are controlled respectively by top gate and back gate respectively for 2D transistors which means two working channel in 2D transistors.

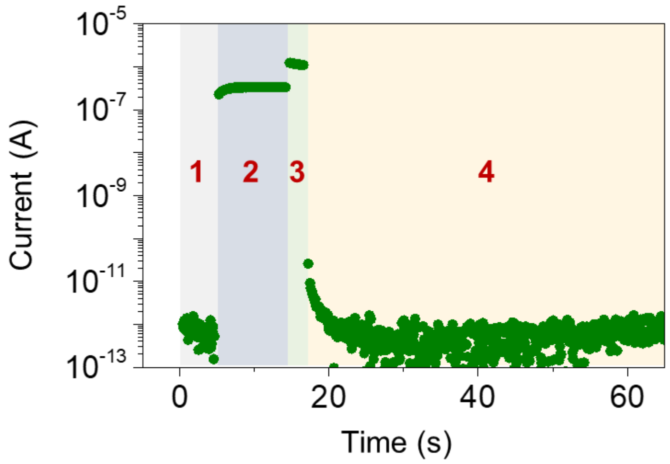

Figure S12. Four operation steps of '0' 'OR' '0' in *in situ* memory. In order to illustrate the operation of *in situ* memory in detail, we have divided the operation process into four steps, corresponding to logic operation (1), waiting time (2), memory operation (3) and data storage monitoring (4). The logic operation has been executed at first in **Part 1**. In this part, logic pulses (± 2 V) are applied from top and back gate respectively. **Part 2** represents the waiting time and there is no action being performed at this moment. The current was read under a V_d bias of 0.5 V. **Part 3** stands for memory operation and memory pulses (± 10 V) are applied from back gate. Nonvolatile memory characteristic was verified before. In this part, the logic result was stored. **Part 4** executes data storage monitoring (no action performed) and the current represents the stored logic result. We could find out that high current state (>10^{-7} A) in **Part 2** turns into low current state (~10^{-12} A) with long retention time in **Part 4** which means the successful storage of logic result.

Supporting Information 1. A detailed comparison with other work

Ohno et al. [13] reported single inorganic synapse with short-term plasticity and long-term potentiation. They focused on a transition from short-term memory to long-term memory by input-pulse repetition time and number. They also demonstrated forgetting curves in short-term memory. However, they could not enable a time-tailoring ability and relation time after each pulse is uncontrollable. In Park's work [14], they also concentrated on short-term to long-term transition based on different pulse number and exhibited one learning experience. The expression about sensory memory and short-term memory is lacking. Kim et al. [10] demonstrated a coexistence of short-term plasticity and long-term potentiation in artificial synapses. They paid attention to the characteristics of short-term memory and short-term to long-term transition. They still suffered from a time-tailoring ability and the demonstrated human memory model is not complete. To sum up, they could only show some features of human memory model for a lack of time-tailoring ability. They preferred to demonstrate a process from short-term memory to long-term memory and retention time for different types of memory is not accurate.

In our work, complete human memory system is implemented in our device, including sensory memory (~millisecond level), short-term memory (~second level) and long-term memory (~minute level). Rich functionalities, such as oblivion, memory transition, memorization-level potentiation and depression, are also demonstrated. Furthermore, our system also has a capability of decision-making and *in situ* data storage.

References

[1] T. Li, G. Du, B. Zhang, Z. Zeng, *Appl. Phys. Lett.* **2014**, *105*, 093107.

[2] K. Cho, W. Park, J. Park, H. Jeong, J. Jang, T.-Y. Kim, W.-K. Hong, S. Hong, T. Lee, *ACS Nano* **2013**, *7*, 7751.

[3] Y. Guo, X. Wei, J. Shu, B. Liu, J. Yin, C. Guan, Y. Han, S. Gao, Q. Chen, *Appl. Phys. Lett.* **2015**, *106*, 103109.

[4] Y. Park, H. W. Baac, J. Heo, G. Yoo, *Appl. Phys. Lett.* **2016**, *108*, 083102.

[5] J. Shu, G. Wu, Y. Guo, B. Liu, X. Wei, Q. Chen, *Nanoscale* **2016**, *8*, 3049.

[6] P. Bousoulas, I. Karageorgiou, V. Aslanidis, K. Giannakopoulos, D. Tsoukalas, *physica status solidi (a)* **2018**, *215*, 1700440.

[7] L. Hu, S. Fu, Y. Chen, H. Cao, L. Liang, H. Zhang, J. Gao, J. Wang, F. Zhuge, *Adv. Mater.* **2017**, *29*, 1606927.

[8] V. K. Sangwan, H.-S. Lee, H. Bergeron, I. Balla, M. E. Beck, K.-S. Chen, M. C. Hersam, *Nature* **2018**, *554*, 500.

[9] Y. Shi, X. Liang, B. Yuan, V. Chen, H. Li, F. Hui, Z. Yu, F. Yuan, E. Pop, H.-S. P. Wong, *Nature Electronics* **2018**, *1*, 458.

[10] M.-K. Kim, J.-S. Lee, *ACS Nano* **2018**, *12*, 1680.

[11] Z. Wang, S. Joshi, S. E. Savel'ev, H. Jiang, R. Midya, P. Lin, M. Hu, N. Ge, J. P. Strachan, Z. Li, *Nat. Mater.* **2017**, *16*, 101.

[12] S. R. Zhang, L. Zhou, J. Y. Mao, Y. Ren, J. Q. Yang, G. H. Yang, X. Zhu, S. T. Han, V. A. Roy, Y. Zhou, *Advanced Materials Technologies* **2018**, 1800342.

[13] T. Ohno, T. Hasegawa, T. Tsuruoka, K. Terabe, J. K. Gimzewski, M. Aono, *Nat. Mater.* **2011**, *10*, 591.

[14] Y. Park, J.-S. Lee, *ACS Nano* **2017**, *11*, 8962.
